# The Clinical Characteristics and Prognostic Nomogram for Head and Neck Cancer Patients with Bone Metastasis

**DOI:** 10.1155/2021/5859757

**Published:** 2021-09-27

**Authors:** Changxing Chi, Zhiyi Fan, Binbin Yang, He Sun, Zengpai Zheng

**Affiliations:** ^1^Department of Anorectal Surgery, The People's Hospital of Pingyang, Wenzhou, Zhejiang, China; ^2^Yunnan Cancer Hospital, Third Affiliated to Kunming Medical University, Kunming, Yunan, China; ^3^Affiliated Hospital of Chengde Medical University, Chengde, Hebei, China; ^4^Wenzhou Medical University, Wenzhou, Zhejiang, China

## Abstract

**Background:**

Head and neck cancer (HNC) is the sixth most common malignancy globally, and many demographics and clinicopathological factors influence its prognosis. This study aimed to construct and validate a prognostic nomogram to predict the prognosis of HNC patients with bone metastasis (BM).

**Methods:**

A total of 326 patients with BM from HNC were collected from the SEER database as the subjects of this study. In a ratio of 7 to 3, patients were randomly divided into training and validation groups. Independent prognostic factors for HNC patients with BM were identified by univariate and multivariate Cox regression analysis. The nomogram for predicting the prognosis was constructed, and the model was evaluated by receiver operating characteristic curves, calibration curves, and decision curve analysis.

**Result:**

The independent prognostic factors for HNC patients with BM included age, primary site, lung metastasis, and chemotherapy. The area under the curve predicting overall survival at 12, 24, and 36 months was 0.768, 0.747, and 0.723 in the training group and 0.729, 0.723, and 0.669 in the validation group, respectively. The calibration curves showed good agreement between the predicted and actual values for overall survival. In addition, the decision curve analysis showed that this prognostic nomogram model has a high clinical application.

**Conclusion:**

This study developed and validated a nomogram to predict overall survival in HNC patients with BM. The prognostic nomogram has high accuracy and utility to inform survival estimation and individualized treatment decisions.

## 1. Introduction

As statistics shows, 430,000 patients are suffering from head and neck cancer (HNC) in the United States in 2016 [1]. Among HNC, squamous cell carcinoma consists of 95% of cases, so most research on HNC focuses on head and neck squamous cell carcinoma, which is the sixth most common neoplasm globally [2, 3]. What is more, HNC has a 1.2–2.8% incidence in distant metastases [4, 5]. Among them, bone metastasis (BM) is the second most common one with a percent of 15–39% [6, 7]. BM can lead to a dismal prognosis and affect patients' quality of life [8, 9]. What is more, it is reported that systemic anticancer treatments such as chemotherapy, immune checkpoint treatment, and targeted therapy can influence the bone microenvironment, leading to BM development [10]. The most common treatment for distant metastases was palliative chemotherapy [11]. Therefore, it is necessary to promote the realization of the prognosis for HNC with BM.

It is reported that primary site and size, tumor grade, and race are risk factors for the development of distant metastasis [6, 12, 13]. Thomas et al. found that the prognosis in the HNC had a significant connection with old age, poorly differentiated tumors, and distant disease at presentation [14]. Research shows that age, sex, race, tumor site, surgery, radiotherapy, and TNM stage can affect long-term overall survival (OS) and cancer-specific survival in head and neck squamous cell carcinoma patients [15]. Although the risk factors for the development of distant metastasis and the prognosis of the HNC have been reported, few researchers pay attention to the prognosis of the HNC with distant metastasis based on big data, not to mention the HNC with BM. The American Joint Committee on Cancer Staging Manual (7th edition) was recommended by the National Comprehensive Cancer Network guidelines to predict the prognosis of HNC patients [16, 17]. But other clinicopathologic factors like age, sex, primary site, and size, race, and treatment can also influence the OS of HNC patients [12, 13], so it is highly needed to build a prognostic prediction model to integrate all significant prognostic factors to accurately predict the survival of the HNC patients with BM. Compared with the TNM staging system, nomogram is a simple predictive tool with graphical representation and higher accuracy for predicting survival, which has more advantages in predicting many cancer clinical results [15, 18]. And there is no relevant research on the nomogram to predict the prognosis of the HNC with BM. Therefore, we developed and validated a nomogram model based on the Surveillance, Epidemiology, and End Result (SEER) database in the present study.

## 2. Methods

### 2.1. Study Population Selection

The patients in the SEER database who were diagnosed with HNC with BM between 2010 and 2015 were included in this study. Because patient information in the SEER database is publicly available and free of charge, institutional review board approval was not required for this study. Inclusion criteria were (1) patients whose only primary site tumor was diagnosed as HNC, (2) patients with BM, and (3) patients with complete clinicopathologic features, demographic data, and survival information. Finally, we screened 326 HNC patients with BM for inclusion in this study. The study population was randomly divided into training and validation groups at a 7 : 3 ratio, and the classification process was performed using R software.

### 2.2. Variable Definitions

The factors in the SEER database that may be relevant to overall survival were enrolled in this study, including age, sex, race, primary site, histological type, grade, T stage, N stage, surgery, radiotherapy, chemotherapy, liver metastasis, brain metastasis, lung metastasis, marital status, and insurance status. Age was changed from a continuous variable to a categorical variable by X-tile software and divided into three groups <47 years, 47–72 years, and >72 years. According to the 7th edition of the American Joint Committee on cancer guidelines, T was divided into T1, T2, T3, and T4, and similarly, N was divided into N0, N1, N2, and N3. In this study, the primary endpoint was overall survival, defined as the time interval between the date of diagnosis and patient death. Regarding marital status, we excluded misleading data on unmarried or domestic partners and then included “unmarried”, “separated”, “single,” and “widowed,” all in the unmarried group. Insurance status is divided into insured and uninsured, with both “insured” and “insured/unspecific” included in the insured group. The primary endpoint of this study was OS, defined as the time interval from the date of diagnosis to the date of patient death.

### 2.3. Statistical Analysis

Prognostic-related factors associated with BM in HNC were identified by univariate Cox regression analysis of related indicators. Subsequently, a multivariate Cox regression analysis was performed for variables with *P* values < 0.05 in the univariate Cox regression analysis to obtain independent prognostic factors for HNC with BM [19]. The prognostic nomogram was developed based on independent prognostic factors using R software's “rms” package. The model performance is divided into two main aspects: discrimination and calibration, which we have validated in the training and validation groups. The calibration curve is a graphical display of calibration accuracy and measures the agreement of predicted probabilities with actual survival outcomes. The discriminant of the model was measured by calculating the area under the receiver operating characteristic curves (AUC), which took values in the range of 0.5–1.0 [20]. To further assess the benefits and advantages of the predictive model, we used decision curve analysis (DCA) [21]. The random grouping, nomogram, calibration curves, AUC, and DCA were composed by R language software (version 4.0.3). In the present study, a *P* value <0.05 (two-sided) indicated statistical significance.

## 3. Results

### 3.1. Baseline Characteristics of HNC Patients with BM

According to our inclusion standards, 326 patients with HNC with BM from the SEER database were included. Of these, 230 patients were enrolled in the training group, and 96 patients were enrolled in the validation group. In the training group, 77.0% of patients were male, 67.8% were white, 68.3% were middle-age (47–72 years old), and 52.2% were unmarried. The most common primary site is the oropharynx (32.2%). The most common T and N stages are T4 (44.3%) and N2 (54.8%), respectively. Among them, there were 14 cases (6.1%) with brain metastasis, 46 cases (20.0%) with liver metastasis, and 69 cases (30.0%) with lung metastasis. The vast majority of patients are insured (89.1%). Regarding therapy, the majority (81.3%) of patients did not receive surgical treatment, 63.9% received chemotherapy, and 54.3% received radiotherapy. The specific demographic and clinical characteristics for all HNC patients with BM are shown in Table 1.

### 3.2. Prognostic Factors for HNC Patients with BM

Univariate and multivariate Cox regression analysis were performed to screen for prognostic factors. After univariate Cox regression analysis, a total of four variables were significantly associated with the prognosis of HNC patients with BM, including age, primary site, lung metastasis, and chemotherapy (Table 2). After controlling for confounding variables with multivariate Cox regression analysis, age, primary site, lung metastasis, and chemotherapy were identified as independent prognostic factors (Table 2).

### 3.3. Development and Validation of a Prognostic Nomogram for HNC Patients with BM

**Figure 1:**
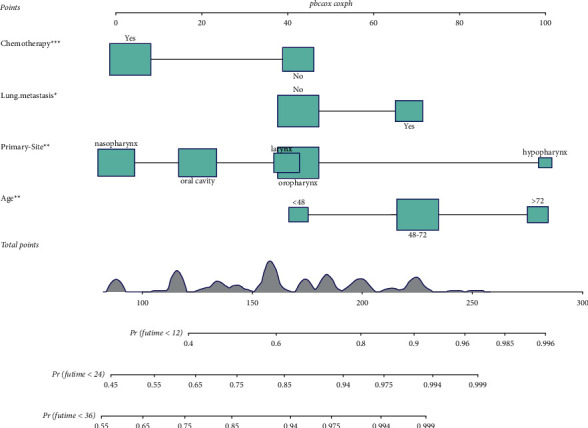
A prognostic nomogram for HNC patients with BM.

**Figure 2:**
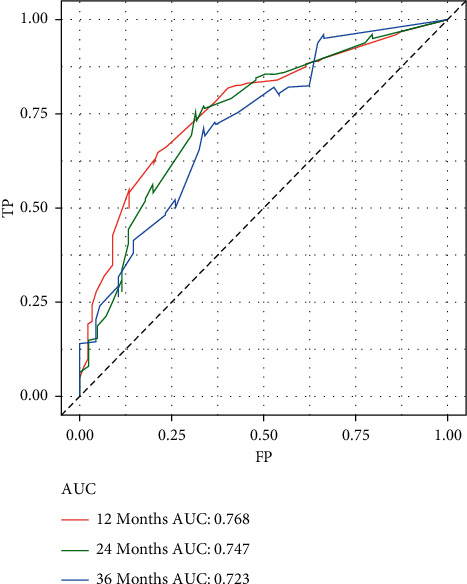
Receiver operating characteristic curves of 12, 24, and 36 months in the training group.

**Figure 3:**
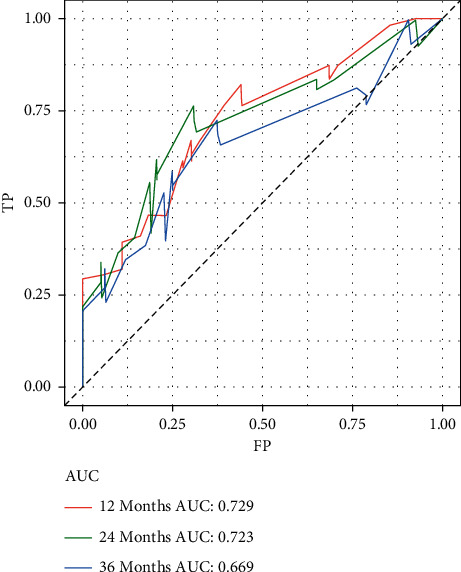
Receiver operating characteristic curves of 12, 24, and 36 months in the validation group.

**Figure 4:**
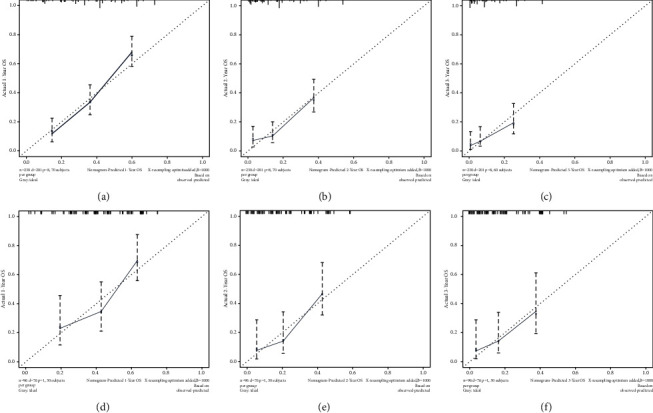
The calibration curves of the prognostic nomogram for the 12-, 24-, and 36-month OS prediction of the training group (A–C) and validation group (D–F). The *x*-axis represents the nomogram-predicted survival rates, whereas the *y* axis represents the actual survival rates.

**Figure 5:**
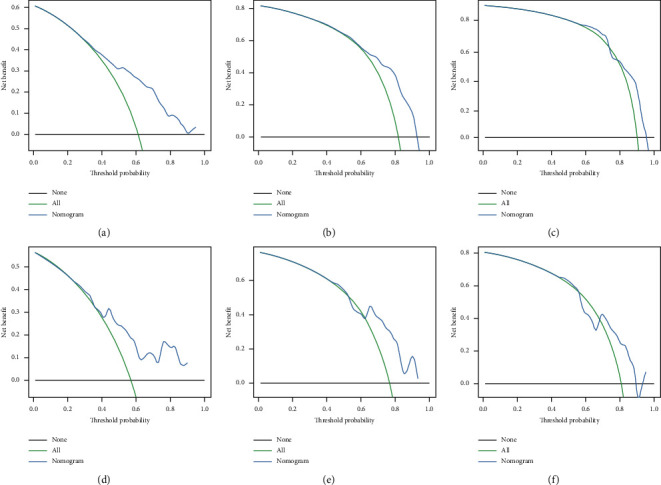
DCA of the prognostic nomogram for the survival prediction of HNC patients with bone metastasis. (a) 12-month survival benefit in the training group. (b) 24-month survival benefit in the training group. (c) 36-month survival benefit in the training group. (d) 12-month survival benefit in the validation group. (e) 24-month survival benefit in the validation group. (f) 36-month survival benefit in the validation group.

Based on the prognostic factors selected in the training group, the prognostic nomogram was established to predict the OS of HNC patients with BM (Figure 1). The primary site has the greatest impact on the prognosis of HNC patients with BM, followed by age. In the prognostic nomogram, values for the individual patient are located along the variable axes, and a line is drawn upward to the points axis to determine the number of points assigned for each variable. The scores for each variable are then summed to calculate an individual's total risk score, and the 12-, 24-, and 36-month OS are estimated visually by drawing a line from the total score axis to the 12-, 24-, and 36-month survival probability axes. The area under the curve of the prognostic model predicting overall survival at 12, 24, and 36 months was 0.768, 0.747, and 0.723 in the training group and 0.729, 0.723, and 0.669 in the validation group, respectively (Figures 2 and 3). As shown in Figure 4, calibration curves were generated to verify the agreement between survival, as predicted by the nomogram, and actual observations. These points are close to a 45-degree diagonal, which indicates that we succeeded in achieving the best agreement between the survival rates predicted by the nomogram and the actual survival rates. Also, the DCA showed that the prognostic nomogram has strong clinical utility (Figure 5).

### 3.4. Stratification of Risk Groups

**Figure 6:**
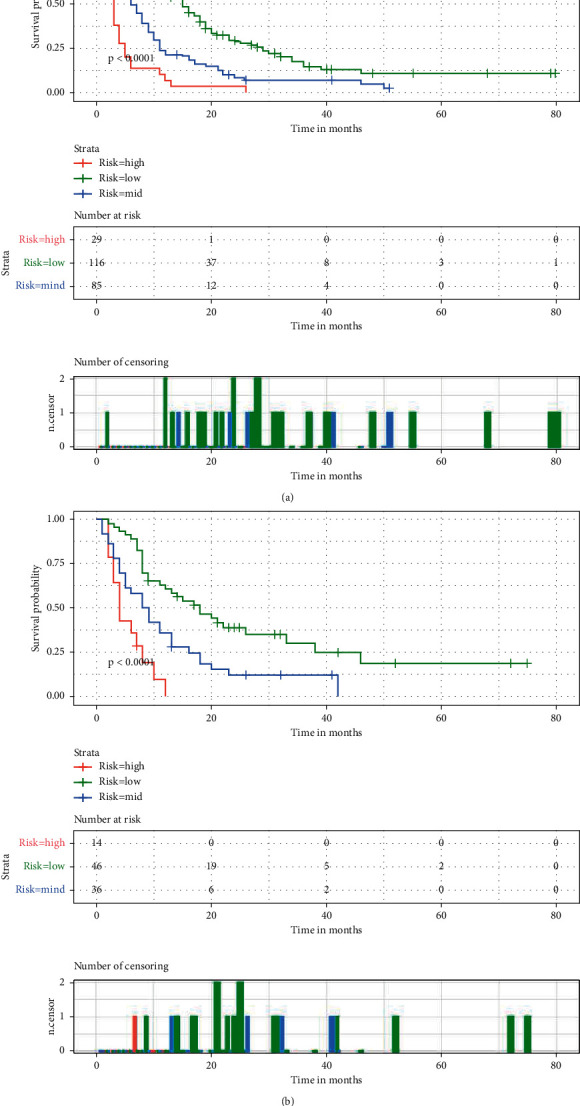
Kaplan–Meier survival analysis of the signature for both the training group (a) and the validation group (b).

The X-tile software was used to classify patients into low mortality risk subgroups, middle mortality risk subgroups, and high mortality risk subgroups. Patients with scores below 157 were classified as a low mortality risk subgroup, those with scores above 220 were classified as a high mortality risk subgroup, and those between 157 and 220 were classified as a middle mortality risk subgroup. As shown in Figure 6, when patients are classified into high mortality risk subgroups, the prognosis is worse than that of patients classified into middle mortality risk subgroups and low mortality risk subgroups.

## 4. Discussion

The prognosis of HNC with BM is poor. Once BM is diagnosed, palliative treatment is the only choice, and the median survival time from BM development is 2–9 months [5, 6, 22]. Therefore, early prophylactic intervention is crucial for patients suffering from HNC with BM. However, there are no studies to predict the prognosis of HNC patients with BM. This study retrospectively analyzed data from the SEER database of patients with HNC with BM. Age, primary site, lung metastasis, and chemotherapy were independent prognostic factors for HNC with BM. Advanced age, primary site in the hypopharynx, presence of pulmonary metastases, and having received chemotherapy were all associated with a higher risk of death. Besides, we developed a clinical prediction model to predict the OS of HNC patients with BM based on the independent prognostic factors.

Suzuki et al. pointed out that neither chemotherapy nor radiotherapy could prolong the OS of HNC patients with BM [23]. In the Cox regression analysis of this study, receiving chemotherapy was an independent risk factor for HNC patients with BM, probably because cells in the bone microenvironment and immune system can promote tumor growth and progression and because the bone is a refuge for cancer cells against anticancer therapies [24]. Bone is an important site for hematopoiesis and is ensured by the bone ecotone, in which different cytokines, growth factors and adhesion molecules play a crucial role [25]. However, the bone ecotone has a close relationship with the tumor microenvironment at the same time. It is the ground for developing several tumor cells, such as primitive hematological cancers and solid metastatic tumors [26]. Previous studies confirmed that cancer cells could disrupt the RANKL/OPG ratio balance in the bone ecotone and increase osteoclast formation, which facilitates bone resorption and metastatic implantation of tumor cells. The process of osteoclast genesis leads to downregulation of immune system pathways in the bone ecotone, creating a vicious circle that promotes tumor bone spread [27]. A retrospective study showed that, compared with patients who did not receive radiotherapy, radiotherapy increased the 5- and 8-year cancer-specific mortality of patients with HNC but reduced the mortality from other causes, thus improving OS at the overall level [15]. Patel et al. showed that surgery and radiation therapy, when used in patients with distant metastatic disease, can improve survival, consistent with our results [28].

Handari et al. reported that head and neck tumors in the hypopharynx at the primary site are more likely to develop distant metastases with a probability of 20.5%–60% and thus have a worse prognosis [6, 13, 29]. Interestingly, in this study, HNC with a primary site in the hypopharynx was indeed more likely to result in higher mortality. Meanwhile, lung metastasis was also an independent risk factor, and when HNC patients with BM had lung metastases, mortality was significantly increased. This result is in line with our usual perception that patients with concurrent multisite metastases always have a worse prognosis than those with single-site metastases. Some studies have shown a survival advantage for married HNC patients and that male oropharyngeal cancer patients may benefit more than women, but in this study, gender and marital status were not associated with OS [23, 30]. Many studies have focused on the prognosis of HNC patients or the risk of developing BM. Still, few studies have focused on the prognosis of HNC with BM, so this article adds clinical reference value to this disease. Studies are suggesting that T and N staging not only affects the incidence of distant metastases in HNC but also affects the prognosis of patients [7, 31]. However, surprisingly, the TMN stage was not a relevant risk factor for the prognosis of HNC patients with BM in the present study.

According to our knowledge, this is the first study to build a nomogram to predict the prognosis of HNC patients with BM based on a large amount of different case data. Nomograms are known as practical tools to quantify risk and maximize prediction accuracy. The prognostic nomogram we constructed accurately estimates the impact of all individual factors on prognosis and enables accurate prediction of OS, our results show. The nomogram can help clinicians develop the surgical, treatment, and follow-up strategies to effectively and individually treat HNC patients with BM. Inevitably, this study also has several limitations. First, some data, such as the location of BM, radiotherapy dose, and selection of chemotherapy drugs, were not recorded by the SEER database and thus were not included in our nomograms. Second, this study was a retrospective cohort study, which may have a lower methodological quality compared to results from randomized trials. Thirdly, only specific information on the four metastatic sites was included, and details of metastases were lacking, such as the number of metastatic foci and the sequence in which the organs became metastatic.

## 5. Conclusion

In brief, we comprehensively identified individual prognostic factors for HNC with BM, including age, primary site, lung metastases, and chemotherapy. This is the first time that a nomogram was proposed to predict the prognosis of HNC with BM. This prognostic nomogram can help clinicians answer patients' clinical inquiries and provide a reference for optimizing treatment plans to improve patients' prognoses.

## Figures and Tables

**Table 1:** Demographic and clinical characteristics of HNSCC patients with bone metastases.

Variables	Training cohort		Validation cohort	
	*N* = 230		*N* = 96	
	*n*	%	*n*	%
*Age*				
<47	34	14.8	14	14.6
47–72	157	68.3	63	65.6
>72	39	17.0	19	19.8
>66	733			

*Race*				
Black	44	19.1	25	26.0
Other	30	13.1	14	14.6
White	156	67.8	57	59.4

*Sex*				
Female	53	23.0	26	27.1
Male	177	77.0	70	72.9

*Primary site*				
Oropharynx	74	32.2	26	27.1
Hypopharynx	7	3.0	7	7.3
Larynx	28	12.2	7	7.3
Nasopharynx	59	25.7	24	25.0
Oral cavity	62	27.0	32	33.3

*Histological types*				
Others	59	25.7	25	26.1
Squamous cell carcinoma	171	74.3	71	73.9

*Grade*				
I	6	2.6	3	3.1
II	60	26.1	25	26.0
III	126	54.8	51	53.1
IV	38	16.5	17	17.7

*T stage*				
T1	35	15.2	10	10.4
T2	41	17.8	20	20.8
T3	52	22.6	24	25.0
T4	102	44.3	42	43.8

*N stage*				
N0	34	14.8	14	14.6
N1	50	21.7	25	26.0
N2	126	54.8	44	45.8
N3	20	8.7	13	13.6

*Surgery*				
No	187	81.3	76	79.2
Yes	43	18.7	20	20.8

*Chemotherapy*				
No	83	36.1	32	33.3
Yes	147	63.9	64	66.7

*Radiotherapy*				
No	105	45.7	35	36.5
Yes	125	54.3	61	63.5

*Liver metastasis*				
No	184	80.0	82	85.4
Yes	46	20.0	14	14.6

*Brain metastasis*				
No	216	93.9	92	95.8
Yes	14	6.1	4	4.2

*Lung metastasis*				
No	161	70.0	66	68.8
Yes	69	30.0	30	31.2

*Insurance status*				
No	25	10.9	13	13.5
Yes	205	89.1	83	86.5

*Marital*				
No	120	52.2	49	51.0
Yes	110	47.8	47	49.0

**Table 2:** Univariate and multivariate Cox regression analysis in HNC patients with bone metastases.

	Univariate Cox analysis				Multivariate Cox analysis			
	HR	95%CI		*P*	HR	95%CI		*P*
*Age*								
<47	1				1			
47–72	2.061	1.319	0.001	0.043	1.496	0.933	2.399	0.095
>72	3.402	2.000	5.788	≤0.001	2.203	1.248	3.888	0.006

*Race*								
Black	1							
Other	0.691	0.412	1.159	0.161				
White	0.904	0.634	1.291	0.580				

*Sex*								
Female	1							
Male	1.150	0.825	1.604	0.409				

*Primary site*								
Oropharynx	1				1			
Hypopharynx	2.357	1.076	5.162	0.032	2.183	0.990	4.813	0.053
Larynx	1.056	0.673	1.657	0.814	0.965	0.613	1.522	0.880
Nasopharynx	0.432	0.293	0.637	≤0.001	0.549	0.363	0.829	0.004
Oral cavity	0.902	0.634	1.283	0.565	0.726	0.499	1.057	0.095

*Histological types*								
Others	1							
Squamous cell carcinoma	1.318	0.950	1.828	0.099				

*Grade*								
I	1							
II	0.902	0.386	2.108	0.812				
III	0.802	0.353	1.825	0.599				
IV	0.540	0.223	1.309	0.173				

*T stage*								
T1	1							
T2	1.539	0.945	2.505	0.083				
T3	1.548	0.971	2.468	0.066				
T4	1.326	0.871	2.019	0.189				

*N stage*								
No	1							
N1	1.255	0.784	2.008	0.344				
N2	1.170	0.771	1.774	0.462				
N3	1.253	0.683	2.299	0.466				

*Surgery*								
No	1							
Yes	0.749	0.524	1.070	0.113				

*Chemotherapy*								
No	1				1			
Yes	0.526	0.396	0.700	≤0.001	0.579	0.422	0.795	≤0.001

*Radiotherapy*								
No	1							
Yes	0.923	0.699	1.218	0.572				

*Liver metastasis*								
No	1							
Yes	1.106	0.789	1.550	0.559				

*Brain metastasis*								
No	1							
Yes	1.179	0.656	2.119	0.582				

*Lung metastasis*								
No	1				1			
Yes	1.552	1.149	2.095	0.004	1.427	1.052	1.936	0.022

*Insurance status*								
No	1							
Yes	0.880	0.565	1.371	0.572				

*Marital*								
No	1							
Yes	0.780	0.591	1.031	0.081				

## Data Availability

The dataset from the SEER database generated and/or analyzed during the current study are available in the SEER dataset repository (https://seer.cancer.gov/).

## Abbreviations

HNC: Head and neck cancer
BM: Bone metastasis
OS: Overall survival
SEER: Surveillance, Epidemiology, and End Results
AUC: Area under the receiver operating characteristic curve
DCA: Decision curve analysis.
